# Large gastroduodenal artery pseudoaneurysm presenting as gastric outlet obstruction: a case report

**DOI:** 10.1093/jscr/rjag130

**Published:** 2026-03-07

**Authors:** Hannah E Hassum, Apoorva Saboo, Aditya Kaushal, Damien Loh, Ee J Ban

**Affiliations:** Acute General Surgery Unit, Department of General Surgery, The Alfred Hospital, 55 Commercial Road, Melbourne, VIC 3004, Australia; Acute General Surgery Unit, Department of General Surgery, The Alfred Hospital, 55 Commercial Road, Melbourne, VIC 3004, Australia; Acute General Surgery Unit, Department of General Surgery, The Alfred Hospital, 55 Commercial Road, Melbourne, VIC 3004, Australia; Acute General Surgery Unit, Department of General Surgery, The Alfred Hospital, 55 Commercial Road, Melbourne, VIC 3004, Australia; Acute General Surgery Unit, Department of General Surgery, The Alfred Hospital, 55 Commercial Road, Melbourne, VIC 3004, Australia

**Keywords:** case reports, visceral artery aneurysm, gastric outlet obstruction

## Abstract

Visceral artery aneurysms are a rare and potentially lethal clinical presentation. They can be asymptomatic or present with large volume gastrointestinal bleeding. Gastric outlet obstruction is a rare clinical presentation for a visceral artery aneurysm. We present a case of a GDA pseudoaneurysm presenting as gastric outlet obstruction and the diagnosis and management of the case. It is important to keep vascular anomalies in mind, particularly when performing endoscopic investigation.

## Introduction

Visceral artery aneurysms and pseudoaneurysms are rare with an estimated incidence of 0.01% to 0.2% [1]. True aneurysms have a complete vascular wall and are caused by the expansion of the entire blood vessel. They are caused by atherosclerosis and hypertension [2]. Pseudoaneurysms are formed by a single layer of fibrous tissue filled with turbulent blood flow. They form from arterial wall damage due to trauma, inflammation (e.g. pancreatitis), autoimmune conditions, infection, or iatrogenic causes [2].

Gastroduodenal artery (GDA) pseudoaneurysm is a rare type of visceral artery aneurysm. The splenic artery is the most common aneurysm (60%), with the GDA accounting for only 1.5% of cases [3]. Visceral artery aneurysms usually affect middle-aged patients between 50 and 58 years old, with a strong male prevalence. For GDA pseudoaneurysms, pancreatitis is the most common cause (47%), followed by ethanol abuse (25%), peptic ulcer disease (17%), and cholecystectomy (3%) [4].

Gastric outlet obstruction (GOO) is a clinical syndrome characterized by physical obstruction at the pylorus, distal stomach, or duodenum, which prevents food and fluids from moving forward into the digestive tract. It arises due to both benign and malignant conditions, the most common causes being peptic ulcer disease, gastric cancer, and periampullary tumours. Benign causes include the formation of strictures due to chronic pancreatitis, anti-inflammatory medications, Crohn’s disease, caustic ingestion, surgical anastomosis scarring, or adhesions [5]. Pseudoaneurysm dilation to the point which it manifests as GOO is an exceedingly rare phenomenon.

## Case presentation

A woman in her 60’s presented to our emergency department with a 4-day history of poor oral intake and vomiting. She denied abdominal pain and was obstipated. Examination findings were abdominal distension with no tenderness or peritonism. Past medical history showed chronic kidney disease secondary to renovascular hypertension, previous stroke, and well-controlled hypertension. Surgical history included elective trapdoor visceral segment aortic endarterectomy for coral reef atherosclerosis on 6 March 2007, which included endarterectomy of both her renal arteries, coeliac artery, and superior mesenteric artery. She reported a history of moderate-large alcohol intake in her 30s since reduced, ex-smoker 44-pack year history, and no recreational drug use.

**Figure 1:**
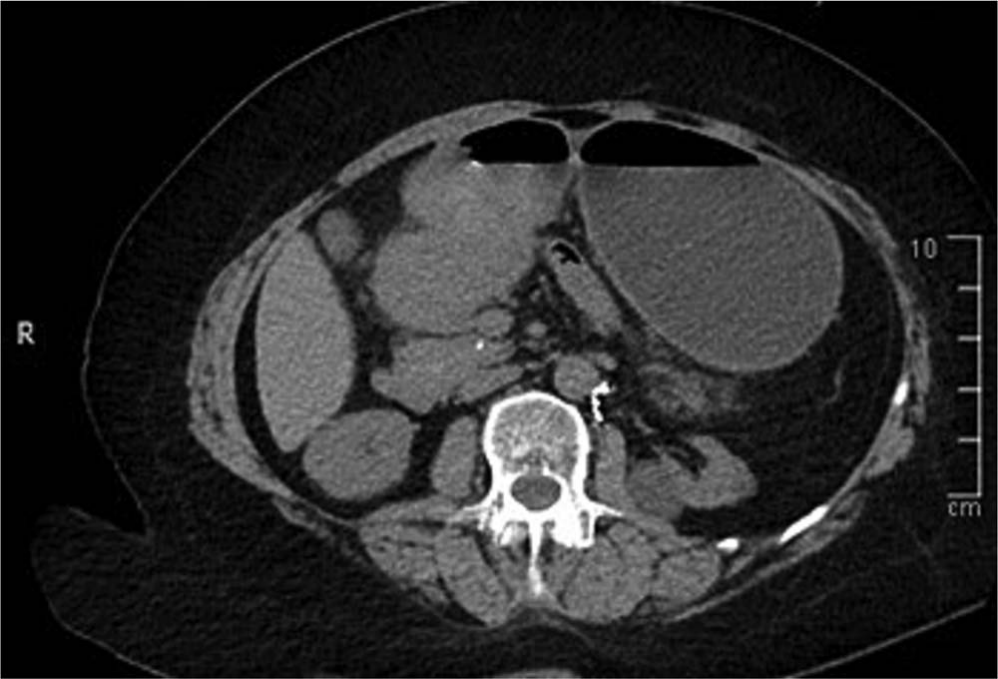
Contrast-enhanced CT of the abdomen. Axial view showing gastric outlet obstruction.

**Figure 2:**
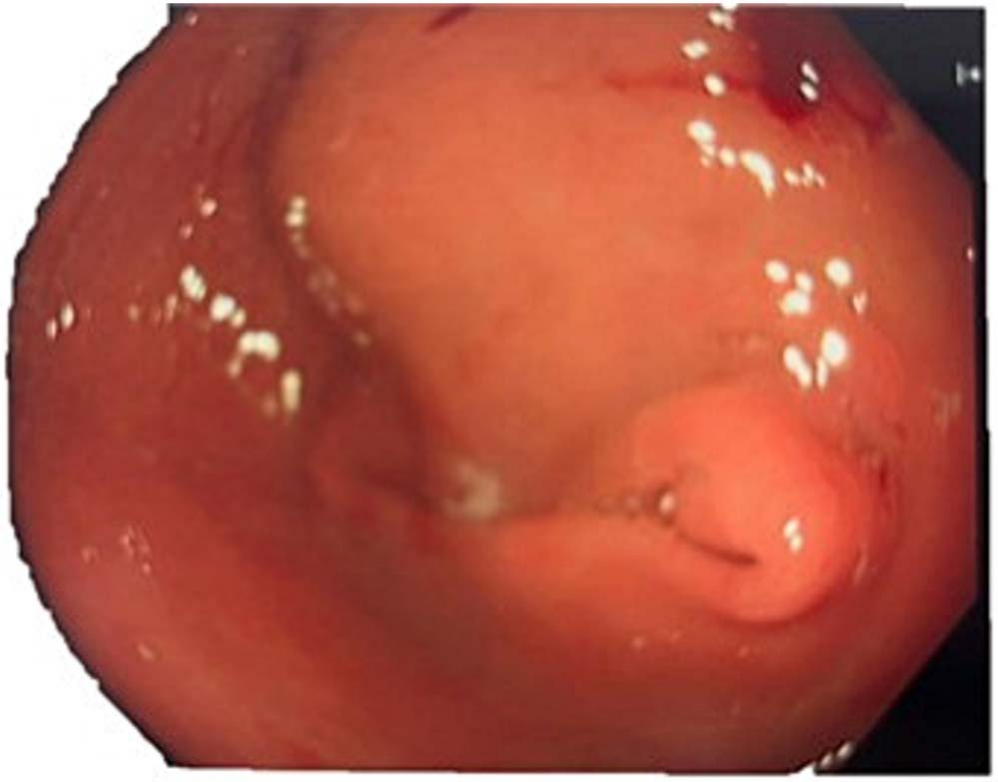
Endoscopic photograph showing large partially obstructing extraluminal mass at the gastric antrum.

**Figure 3:**
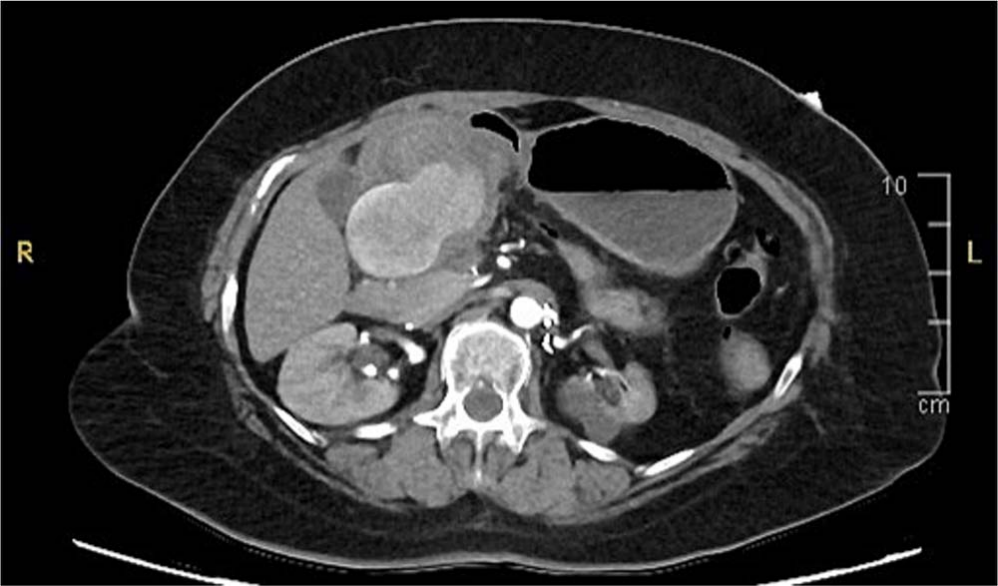
Contrast-enhanced CT angiography of the abdomen. Axial view showing a large 6.2 × 4.2 × 7.0 cm pseudoaneurysm arising from the gastroduodenal artery.

**Figure 4:**
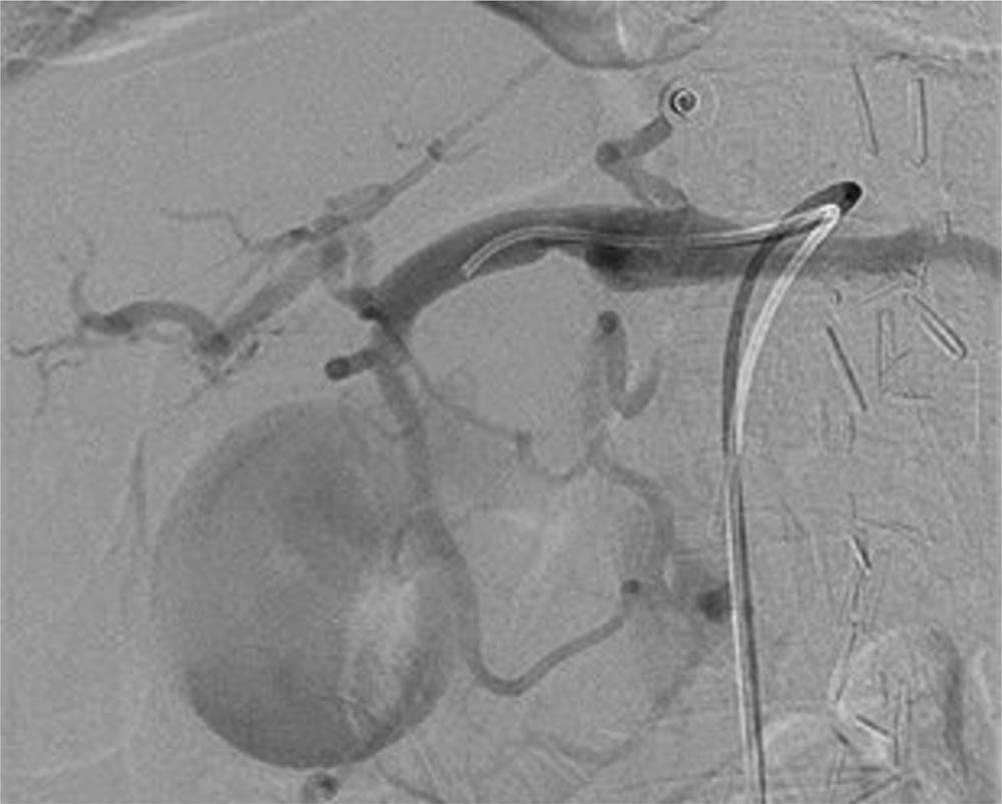
Intraoperative photograph of successful coil angioembolization of the pseudoaneurysm.

A computed tomography (CT) of the abdomen with IV portal venous contrast demonstrated proximal gastric dilatation due to a 7.6 cm gastric pyloric mass concerning for malignancy (Fig. 1). Other findings included small volume pancreatic parenchymal calcifications with no ductal dilatation. She was decompressed via nasogastric tube and planned for gastroscopy and feeding tube insertion. Gastroscopy demonstrated a partially obstructing extraluminal mass at the gastric antrum, able to be traversed to the second part of the duodenum (Fig. 2). A nasojejunal feeding tube was placed and the nasogastric tube was left in for decompression. An endoscopic ultrasound the following day demonstrated Doppler signal in the mass, confirmed to be a large 6.2 × 4.2 × 7.0 cm pseudoaneurysm arising from the GDA on a subsequent CT mesenteric angiogram (Fig. 3). Coil angioembolization of the pseudoaneurysm was successfully done via common femoral access (Fig. 4). She was commenced on NJ feeding and supplemental parenteral nutrition. She recovered well and was discharged on puree diet. Repeat imaging at 4 weeks demonstrated resolution of the pseudoaneurysm and returned to normal diet 6 weeks post presentation.

## Discussion

This is an unusual presentation for a GDA pseudoaneurysm as gastric outlet obstruction. Amongst the major risk factors predisposing patients to GDA pseudoaneurysm, our patient had a history of smoking, previous alcohol intake, and radiological evidence of chronic pancreatitis with no reported history of pancreatitis. However, pancreatitis is the leading cause of GDA pesudoaneurysms [4].

Gastric outlet obstruction due to GDA psuedoaneurysms is rare with only five cases in the literature. Three were successfully managed with coil angioembolization [6–8]. In one case the GDA pseudoaneurysm was treated with N-butyl-2-cyanoacrylate [9]. In the first case of GDA pseudoaneurysm causing GOO described in the literature (2001), the patient unfortunately passed away prior to intervention [10].

Most visceral artery aneurysms are usually asymptomatic and found on routine abdominal imaging. When GDA pseudoaneurysms do present symptomatically, they do so dramatically, with large volume bleeding as the most common presentation [4]. A ruptured GDA pseudoaneurysm carries a 40%–70% morality rate [11]. An unruptured GDA pseudoaneurysm commonly presents as abdominal pain, nausea, and vomiting from compression of gastrointestinal tract, as in our patient. A GDA pseudoaneurysm can present as obstructive jaundice due to the proximity between GDA and the bile duct [2]. The Society for Vascular Surgery clinical practice guidelines (2020) recommends intervention for GDA pseudoaneurysm of any size, given the risk of rupture [12].

Several management options are available for GDA pseudoaneurysms, including endovascular coil embolization, stent-graft placement, and surgical repair. The first-line treatment is endovascular coil embolization. Endovascular techniques have emerged as the gold standard due to their minimally invasive technique, reduced morbidity, and shorter recovery time compared to open surgery [13]. The overall complications from endovascular embolization are < 5% [13]. Our successfully underwent endovascular coiling, which treated her GOO and obliterated her pseudoaneurysm.

If coil embolization is not feasible, flow-diverting, multilayered stents, covered stenting or stent-assisted coil embolization can be considered; however further evidence is needed to be recommended as a primary treatment modality [12]. Stent-graft placement can preserve arterial flow but may not be feasible in tortuous anatomy or small-caliber vessels, and carries a risk of endoleak or stent migration.

In unstable patients or those refractory to less invasive methods, open surgical techniques including revascularization, vessel ligation, and aneurysmal sac exclusion are required [11]. Whilst definitive in their management, open techniques carry a higher morbidity and mortality and are reserved for those who fail less invasive interventions.

## Conclusion

GDA pseudoaneurysm is a rare and potentially lethal consequence of atherosclerotic disease and chronic pancreatitis. In this case, the patient’s symptoms initially mimicked gastric malignancy and were investigated as such. It is important to keep vascular anomalies in mind, particularly when performing endoscopic management, as an inadvertent biopsy of the pseudoaneurysm could have had catastrophic consequences for the patient. It would be paramount to consider such a differential diagnosis for a patient with atherosclerotic disease and/or pancreatitis. Furthermore, coil angioembolization is a successful and well tolerated intervention for such cases.
